# A reversible *Renilla* luciferase protein complementation assay for rapid identification of protein–protein interactions reveals the existence of an interaction network involved in xyloglucan biosynthesis in the plant Golgi apparatus

**DOI:** 10.1093/jxb/eru401

**Published:** 2014-10-18

**Authors:** Christian H. Lund, Jennifer R. Bromley, Anne Stenbæk, Randi E. Rasmussen, Henrik V. Scheller, Yumiko Sakuragi

**Affiliations:** ^1^University of Copenhagen, Department of Plant Biology and Biotechnology, Frederiksberg, DK-1871, Denmark; ^2^Joint BioEnergy Institute, Feedstocks Division, Emeryville, CA 94608, USA; ^3^Physical Biosciences Division, Lawrence Berkeley National Laboratory, Berkeley, CA 94720, USA; ^4^Department of Plant and Microbial Biology, University of California, Berkeley, CA 94720, USA

**Keywords:** *Arabidopsis thaliana*, glycosyltransferase, Golgi apparatus, *Nicotiana benthamiana*, plant cell wall, polysaccharides, protein–protein interaction, *Renilla* luciferase, type II membrane protein, xyloglucan.

## Abstract

A growing body of evidence suggests that protein–protein interactions (PPIs) occur amongst glycosyltransferases (GTs) required for plant glycan biosynthesis (e.g. cell wall polysaccharides and *N*-glycans) in the Golgi apparatus, and may control the functions of these enzymes. However, identification of PPIs in the endomembrane system in a relatively fast and simple fashion is technically challenging, hampering the progress in understanding the functional coordination of the enzymes in Golgi glycan biosynthesis. To solve the challenges, we adapted and streamlined a reversible *Renilla* luciferase protein complementation assay (*R*luc-PCA), originally reported for use in human cells, for transient expression in *Nicotiana benthamiana.* We tested *R*luc-PCA and successfully identified luminescence complementation amongst Golgi-localizing GTs known to form a heterodimer (GAUT1 and GAUT7) and those which homooligomerize (ARAD1). In contrast, no interaction was shown between negative controls (e.g. GAUT7, ARAD1, IRX9). *R*luc-PCA was used to investigate PPIs amongst Golgi-localizing GTs involved in biosynthesis of hemicelluloses. Although no PPI was identified among six GTs involved in xylan biosynthesis, *R*luc-PCA confirmed three previously proposed interactions and identified seven novel PPIs amongst GTs involved in xyloglucan biosynthesis. Notably, three of the novel PPIs were confirmed by a yeast-based split-ubiquitin assay. Finally, Gateway-enabled expression vectors were generated, allowing rapid construction of fusion proteins to the *R*luc reporters and epitope tags. Our results show that *R*luc-PCA coupled with transient expression in *N. benthamiana* is a fast and versatile method suitable for analysis of PPIs between Golgi resident proteins in an easy and mid-throughput fashion *in planta.*

## Summary

A reversible *Renilla* luciferase protein complementation assay for rapid identification of protein–protein interactions revealed the existence of an interaction network involved in xyloglucan biosynthesis in the Golgi apparatus in plants.

## Introduction

In eukaryotes, biosynthetic machineries of extracellular matrix polysaccharides (e.g. cell wall polysaccharides, proteoglycans) and *N-* and *O*-protein glycosylation are located in the endomembrane system, particularly in the Golgi apparatus. It is known in plants, yeast, and mammals that the Golgi-localization and function of some of glycan biosynthetic enzymes, including glycosyltransferases (GTs), are regulated through protein–protein interactions (PPIs) (de Graffenried and Bertozzi, 2004; Jungmann and Munro, 1998; McCormick *et al.*, 2000; Schoberer *et al.*, 2013; Tu and Banfield, 2010). A growing body of evidence suggests that functions of some GTs and other proteins involved in plant cell wall biosynthesis are also controlled by PPIs (Oikawa *et al.*, 2013). In dicots, pectins and hemicelluloses are two main classes of plant cell wall polysaccharides, the biosynthesis of which occurs in the Golgi apparatus (Driouich *et al.*, 2012; Dupree and Sherrier, 1998), and which play pivotal roles in providing physical support to the cell wall and during plant growth, development, reproduction, and in response to environmental stimuli (Bellincampi *et al.*, 2014; Scheller and Ulvskov, 2010; Wolf *et al.*, 2009). These polysaccharides also present a rich renewable source of biomass for a wide range of industrial processes including production of food, fuel, and fibres (Carpita, 2012; Pauly and Keegstra, 2008). Impressive progress has been made during the past decade in the identification of genes and enzymes involved in pectin and hemicellulose biosynthesis; however, mechanisms of their functional control by PPIs remain largely elusive, owing in part to technical limitations as described below.

In *Arabidopsis thaliana*, the following PPIs have been experimentally demonstrated for GTs involved in cell wall biosynthesis. The term ‘GT’ is used for proteins that are classified as GTs by the Carbohydrate Active Enzyme database (http://www.cazy.org) based on phylogenetic relatedness even though some of these proteins, such as GALACTURONOSYLTRANSFERASE7 (GAUT7) may not have glycosyltransferase activity (Atmodjo *et al.*, 2011). Of note, heterooligomerization of GAUT1 and its homologue, GAUT7, in biosynthesis of pectic homogalacturonan (Atmodjo *et al.*, 2011); hetero- and homo-oligomerization of the putative arabinosyltransferases ARABINAN DEFICIENT1 (ARAD1) and ARAD2 in pectic arabinan biosynthesis (Harholt *et al.*, 2012; Søgaard *et al.*, 2012); and homo- and heterooligomerization of XYLOSYLTRANSFERASE1 (XXT1), XXT2, XXT5, and CELLULOSE-SYNTHASE LIKE C4 (CSLC4) in hemicellulosic xyloglucan (XyG) biosynthesis (Chou *et al.*, 2012) have been demonstrated. In wheat, a high-molecular weight complex involving GTs belonging to the GT families 43, 47, and 75 has been identified in glucurono(arabino)xylan biosynthesis (Zeng *et al.*, 2010). In addition, it has been proposed that UDP-arabinose mutases, also known as REVERSIBLY GLYCOSYLATED POLYPEPTIDEs (RGPs), required for the interconversion of UDP-arabinopyranose to UDP-arabinofuranose, are retained at the cytosolic face of the Golgi membrane by PPIs for efficient supply of the substrate to the Golgi lumen (Konishi *et al.*, 2007; Rautengarten *et al.*, 2011). Precise biological/biochemical functions of these PPIs are only beginning to be understood. For instance, GAUT1 undergoes proteolytic processing in the endomembrane system, which renders the C-terminal catalytic domain of GAUT1 secretable, whereas GAUT7, which does not seem to undergo proteolytic processing, anchors the secretable C-terminal domain of GAUT1 in the Golgi lumen where homogalacturonan biosynthesis occurs (Atmodjo *et al.*, 2011). Given the structural complexity of polysaccharides, PPIs among GTs, modifying enzymes (e.g. methyl- and acetyltransferases, nucleotide-sugar converting enzymes) and other functional proteins (e.g. transporters and chaperones) are likely to occur and may play pivotal roles in the regulation of biosynthesis of these polymers (Oikawa *et al.*, 2013). Analysis of PPIs among integral membrane proteins in the Golgi apparatus is technically challenging because many of these proteins are often in low abundance and isolation of protein complexes requires careful choice and optimization of conditions (e.g. detergents, buffers, chromatographic resins) for each individual PPI investigated. *In situ* binary PPI assays, such as bimolecular fluorescence complementation (BiFC), and Förster resonance energy transfer (FRET) are non-invasive and can facilitate identification of PPIs in living tissues, and in addition can provide information about the subcellular localization of PPIs in cells. However, these techniques have their caveats. In BiFC, the split halves of the fluorophores irreversibly assemble, causing a high rate of false-positive interactions (Magliery *et al.*, 2005; Søgaard *et al.*, 2012; Zamyatnin *et al.*, 2006). Although BiFC has been successfully used to aid identification of PPIs among the Golgi-localizing GTs in plants and mammals (Chou *et al.*, 2012; Hassinen *et al.*, 2010), it also requires use of a flow cytometer, an expensive and maintenance-intensive instrument, for high-confidence identification of PPIs. Whereas FRET offers a lower false positive rate, it has a low signal-to-noise ratio and requires additional data processing and an expensive instrumental setup (Piehler, 2005). The split-ubiquitin assay in yeast (Stagljar *et al.*, 1998) is widely used for PPIs among membrane proteins. Ubiquitin is split into two fragments, the N-terminal ubiquitin fragment (Nub) and the C-terminal ubiquitin fragment (Cub). The native NubI, with “I” being isoleucine at position 13, interacts irreversibly with Cub and is used as a positive control, whereas NubG, with “G” being glycine replacing the isoleucine, interacts reversibly with Cub and is used for the interaction assay (Johnsson and Varshavsky, 1994). Cub is fused to a synthetic transcription factor (TF) (protein A–LexA–VP16), and when reconstituted with NubI or NubG the C-terminus of Cub is cleaved by cytosolic ubiquitin-specific proteases releasing the synthetic transcriptional factor that subsequently initiates transcription of reporter genes. The split-ubiquitin assay is powerful as it allows high-throughput screening of PPIs amongst membrane-bound proteins, and has been successfully used in characterisation of the cellulose synthase complex in *Arabidopsis* (Timmers *et al.*, 2009). However, as plant proteins are expressed in a non-native system, misfolding and mislocalization can result in a relatively high rate of false-negative interactions (Oikawa *et al.*, 2013).

In this article, we present a successful adaptation of a reversible *Renilla* luciferase complementation assay (*R*luc-PCA), previously reported in human cells (Stefan *et al.*, 2007), for screening of PPIs among Golgi-localizing proteins *in planta*. Luciferase-based PCA offers a superb signal-to-noise ratio and maintains reversibility of PPIs (Stefan *et al.*, 2007). *Agrobacterium tumefaciens*-mediated transient transfection of *Nicotiana benthamiana* was used to express proteins of interest (POI) fused with the N- and C-terminal human-codon optimized *Renilla* luciferase (h*R*luc) fragments in Gateway-enabled expression vectors. Co-transfection of Agrobacterial strains carrying different POI-h*R*luc constructs allowed versatility in choice of binary interaction assay to be performed. To strengthen the versatility of the system, compatible Gateway expression vectors for the yeast split-ubiquitin assay were generated. The assay is easy, robust, and requires standard laboratory equipment. Furthermore, using *R*luc-PCA enabled successful identification of novel candidates for PPIs amongst XyG biosynthetic enzymes.

## Materials and methods

### Generation of h*R*luc and ST–h*R*luc

h*R*luc was reconstituted from the two h*R*luc fragments (a gift from S.W. Michnick, University of Montreal) by USER fusion (Geu-Flores *et al.*, 2007; Nour-Eldin *et al.*, 2006). h*R*luc fragment [F1] was amplified from PKACat.h*R*luc-F[1] (Stefan *et al.*, 2007) using primers USERF1 F and USERF1 R. Fragment [F2] was amplified from PKACat.h*R*luc-F[2] (Stefan *et al.*, 2007) using USERF2 F and USERF2 R. The two products were combined and inserted into pCambia3300u (Geu-Flores *et al.*, 2007; Nour-Eldin *et al.*, 2006) by USER fusion. Gateway entry clones were produced for both the N-terminus of rat sialyltransferase (ST) combined with h*R*luc (ST–h*R*luc) and h*R*luc in pDONR^TM^/Zeo by BP recombination. An *att*B flanked h*R*luc was amplified using primers *att*B1Luc F and *att*B2Luc R. An *att*B flanked ST–h*R*luc was produced by overlap PCR. ST was amplified from Yn-TMD (Søgaard *et al.*, 2012) using primers *att*B1ST F and LucST R. h*R*luc was amplified using primers STLuc F and *att*B2Luc R. Products were combined and *att*B1ST F and *att*B1Luc R primers used to amplify the ST–h*R*luc chimera. Primer sequences are detailed in Supplementary Table S1. Constructs for measurement of activity of the ST–h*R*luc fusion protein and h*R*luc were produced without C-terminal epitope fusions by recombination with pEarleygate100 (Earley *et al.*, 2006). Constructs for localization of the ST–h*R*luc fusion protein and h*R*luc were produced by LR recombination with pEarleygate101 to produce C-terminal YFP fusions.

### Transient expression in *N. benthamiana*

Transient expression in *N. benthamiana* was performed as described by Sakuragi *et al.* (2011) using *Agrobacterium tumefaciens* GV3101 as a bacterial host and included the co-infiltration of the viral silencing suppressor p19 (Voinnet *et al.*, 2003). Transient expression of fusion proteins was carried out in 4-week-old *N. benthamiana* plants grown under a 16h photoperiod at 26/24°C (day/night), 60% humidity and light intensities of 115–150 µE m^–2^ s^–1^. Each *A. tumefaciens* strain was infiltrated at a final OD_600nm_ of 0.2, unless stated otherwise, and that harbouring p19 at OD_600_ 0.05. Infiltrated plants were returned to the same growth conditions for 72h before harvest of material.

### Fluorescence confocal microscopy

ST–h*R*luc–YFP and the Golgi marker α-mannosidase–CFP (Nelson *et al.*, 2007) were co-infiltrated into *N. benthamiana* to confirm targeting of ST–h*R*luc to the Golgi apparatus. Abaxial epidermal sections from leaves 72h post infiltration were prepared. A Zeiss LSM 710 confocal microscope equipped with Argon and InTune lasers was used for confocal laser-scanning microscopy. All images were obtained with a 0.9NA 40X air objective using the Zen software package (Carl Zeiss Inc., Oberkochen, Germany). Emission was collected at 463 to 484nm (CFP) and 521 to 572nm (YFP), laser lines 405nm and 514nm. The pinhole diameter was set at 1 airy unit. Image analysis and processing (scale bar, brightness, and contrast) used ImageJ (Version 1.6r).

### Construction of ph*R*luc[F1] and ph*R*luc[F2] vectors

Gateway compatible ph*R*luc[F1] and ph*R*luc[F2] vectors were generated by USER cloning of overlap PCR amplicons into pCambia3300u downstream of a CaMV 35S promoter. pEarleygate101 was used as a template to amplify the Gateway cassette, PKACat.h*R*luc-F[1] for h*R*luc [F1] and PKACat.h*R*luc-F[2] for h*R*luc [F2]. In addition, a C-terminal HA epitope was added to h*R*luc-[F1] and a FLAG epitope to h*R*luc [F2] by inclusion in the reverse primer sequence. All primers used are detailed in Supplementary Table S1. The amplicons for fusion were amplified using the products of USER GW F and LucF1GW R, and GWLucF1 F and USERLucF1HA R for ph*R*luc[F1] and USERGW F and LucF2GW R combined with GWLucF2 F and USERLucF2FL R for ph*Rl*uc[F2]. The final cassettes were amplified from a mixture of the two fragments with USER site flanked primers.

### Gateway enabled DUALmembrane system vectors

pPR3-N and pBT3-N (Dualsystems Biotech AG, Schlieren, Switzerland) were used to create Gateway destination vectors by ligation of a Gateway cassette by the *Sfi*I site downstream of the NubG and Cub, thereby generating pPR3-GW and pBT3-GW, respectively. The Gateway cassette was amplified from pEarleygate101 using primers SfiGW F and SfiGWStop R to include a stop codon in frame to the 3’ of the *att*R2 site.

### Generation of HG, XyG, and xylan-related GT *R*luc-PCA constructs

Coding sequences lacking stop codons of GAUT1 (At3g61130.1), GAUT7 (At2g38650.1), ARAD1 (At2g35100.1), IRREGULAR XYLEM9 (IRX9, At2g37090.1), XXT5 (At1g74380.1), MURUS3/KA- TAMARI1 (MUR3) (At2g20370.1) and FUCOSYLTRANSFERA- SE1 (FUT1, At2g03220.1) were inserted into pDONR^TM^/Zeo by BP recombination (Invitrogen, Carlsbad, CA, USA), primers detailed in Supplementary Table S1. Coding sequences of XXT1 (At3g62720.1), XXT2 (At4g02500.1), CSLC4 (At3g28180.1), IRX9-LIKE (IRX9-L, At1g27600.1), IRX10 (At1g27440.1), IRX10-L (At5g61840.1), IRX14 (At4g36890.1) and IRX14-L (At5g67230.1) in pDONR223 lacking stop codons were obtained from the JBEI GT collection (Lao *et al.*, 2014). In-frame fusions into ph*R*luc[F1] and ph*R*luc[F2] vectors were made by LR recombination (Invitrogen, Carlsbad, CA, USA). Additionally, the 35S–ARAD1–cMyc construct, a gift from J. K. Jensen (Michigan State University, USA) was used as the competitor in the competition assay.

### Reversible *Renilla* luciferase protein complementation assay

Three leaf discs (Ø 0.5cm), one from each of three infiltrated leaves, were punched out and pooled into tubes containing 200 µl ice cold assay buffer [0.5M NaCl, 0.1M potassium phosphate pH 7.4, 1mM EDTA, 0.02% (w/v) BSA supplemented with protease inhibitors (cOmplete EDTA-free protease inhibitor, Roche, Basel, Switzerland)] and a chrome ball (Ø 3mm). The plant material was macerated in a mixer mill (Retsch MM301, Haan, Germany) at 25–30 Hz for 1min. Samples were kept on ice whenever possible. Of each sample, 100 µl was transferred to a Nunc black 96-well plate (Thermo Scientific, Rockford, IL, USA). Coelenterazine-h (Biosynth AG, Staad, Switzerland) was added to a final concentration of 10 µM to each well by an automated injector and bioluminescence measured for 30 s immediately after addition using a luminometer (Berthold TriStar^2^ LB 942, Berthold, Bad Wildbad, Germany). For each PPI tested, three independent samples, each comprised of a pool of three independent leaf discs, were assayed. The experiment was repeated three times with independent transfection of *N. benthamiana.* Means of the RLU values derived from the three independent experiments were transformed to the Log_10_ scale, which were used for statistical evaluation by Student′s t-test (independent test with two tails) for evaluation of the difference from the Log_10_-transformed RLU value obtained for samples expressing p19 alone.

### Immunoblotting

Pooled leaf discs as described above were either homogenised directly in 100 µl Laemmli buffer or were macerated in the *R*luc-PCA assay buffer and Laemmli buffer added. The samples were boiled for 5min and cooled on ice. Ten microliters of the homogenate were separated on a 12% 1-mm thick polyacrylamide gel (Criterion™ XT Bis-Tris precast polyacrylamide gel, Biorad, Hercules, CA, USA) in 1× XT-MOPS buffer (Biorad, Hercules, CA, USA). Proteins were transferred to a nitrocellulose membrane and probed with primary and secondary antibodies. Antibodies were diluted in PBS-T 1% (w/v) skimmed milk powder as the following: rabbit α-HA (Sigma-Aldrich, St. Louis, MO, USA), 1:500; swine α-rabbit HRP-conjugate (Dako, Glostrup, Denmark), 1:1700; mouse α-FLAG M2 (Sigma-Aldrich), 1:1000; rabbit α-mouse HRP-conjugate (Dako, Glostrup, Denmark), 1:2000; mouse α-cMyc 9E10 (Sigma-Aldrich), 1:1000, where skimmed milk powder was omitted. Detection was performed with SuperSignal West Dura chemiluminescent substrate (Thermo Scientific, Rockford, IL, USA).

### Yeast split-ubiquitin assay

The split-ubiquitin assay was performed in yeast strain NMY51 using pBT3-N/pBT3-GW and pPR3-N/pPR3-GW vectors (Dualsystems Biotech AG, Schlieren, Switzerland). The coding sequences of tested GTs were PCR amplified using primers detailed in Supplementary Table S1, and ligated into pBT3-N and pPR3-N (Dualsystems Biotech AG, Schlieren, Switzerland) at the *Sfi*I restriction site. The coding sequence of FUT1 was inserted in-frame into pBT3-GW and pPR3-GW by LR recombination. The plasmids were introduced in pairs into NMY51 by LiAc transformation (Gietz and Woods, 2002). Transformants were selected on SD-Leu-Trp and strains carrying both vectors were grown to OD_546_ of 1.5. Serial dilutions (from 1–1000 fold) were spotted on SD-His-Leu, SD-His-Leu-Trp and SD-His-Leu-Trp-Ade plates. Growth on SD-His-Leu-Trp-Ade plates was scored as an indication of interaction. Yeast growing on SD-His-Leu plates were tested for β-galactosidase activity using the X-gal overlay assay (Obrdlik *et al.*, 2004).

## Results and discussion

### The choice of luciferase-based PCA system for analysis of PPIs in the Golgi lumen

**Fig. 1.**
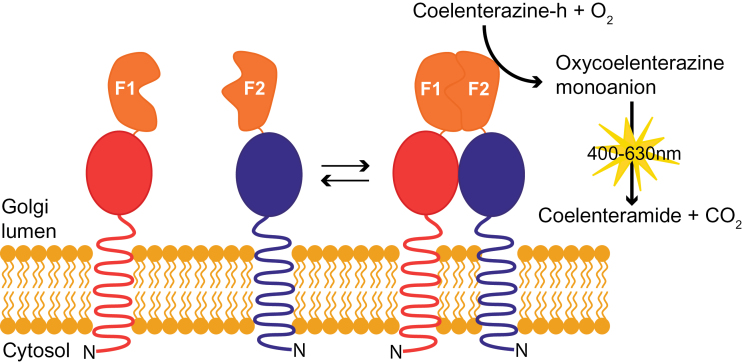
Schematic representation of the reversible *Renilla* luciferase protein complementation assay (*R*luc-PCA) to study Golgi lumenal protein interactions. Membrane proteins with a type II membrane topology, spanning the membrane once with the N-terminus (N) in the cytosol and a lumenal C-terminus, are shown fused to the N-terminal domain (F1) and C-terminal domain (F2) of human-codon optimized *Renilla* luciferase (h*R*luc). Arrows denote the dynamics of the protein interaction, the coupling and decoupling of the two domains of h*R*luc. (This figure is available in colour at *JXB* online.)

Before this study, two versions of luciferase-based PCA had been developed for study of PPIs among cytosolic proteins *in planta*. Firefly luciferase was used to successfully detect PPIs amongst cytosolic proteins in *N. benthamiana* (Gehl *et al.*, 2011). However, this system is unsuitable for PPI assays in the Golgi lumen because of the absence of ATP in this compartment. A luciferase from sea pansy (*Renilla reniformis*; *R*luc) does not require ATP for its catalytic action and has been successfully used for *in vivo* detection of PPIs amongst cytosolic proteins in *Arabidopsis* protoplasts (Fujikawa and Kato, 2007; Kato and Jones, 2010). This system also integrated a Gateway- and Cre-loxP-enabled vector cloning system, allowing high-throughput cloning and screening of PPIs *in planta*. However, reversibility of the association between the two *R*luc fragments (amino acid residues 1–299, N-terminal fragment; residues 299–310, C-terminal fragment) has not yet been experimentally demonstrated. A human-codon optimized *R*luc PCA with structure-based design of fragments (amino acid residues 1–110, N-terminal fragment [F1]; residues 111–310, C-terminal fragment [F2]) has been developed for use in human cell line HEK293T and Chinese hamster ovary cells (Stefan *et al.*, 2007). Notably, the reversible reconstitution of the two fragments has been experimentally demonstrated. The reversibility of the system is particularly important for an assay system dealing with endomembrane proteins because their diffusion is limited in a restricted two-dimensional space. As a consequence there would be a considerably higher frequency of false-positive interaction should the two fragments irreversibly assemble. Therefore, we have used *R*luc-PCA for the subsequent experiments and used *N. benthamiana* as expression host owing to its ease of transfection and efficient expression of transient proteins with minimal handling compared with *Arabidopsis* protoplast based assays. A schematic representation of *R*luc-PCA adapted for a Golgi PPI assay is shown in Fig. 1.

### Assay of h*R*luc activity in the Golgi apparatus of *N. benthamiana*

We placed the h*R*luc fragments on the carboxy (C) termini of the POIs because amino (N) terminal tagging of integral membrane proteins may affect their membrane protein topologies (Søgaard *et al.*, 2012). Furthermore, there is precedence for post-translational proteolytic processing that cleaves the N-terminal domain from the C-terminal domain that contains features required for PPIs (Atmodjo *et al.*, 2011).

**Fig. 2.**
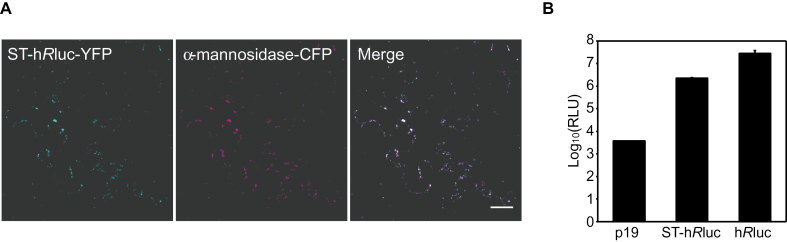
Localization and activity of Golgi lumenal localized human-codon optimized *Renilla* luciferase (h*R*luc). Rat sialyltransferase transmembrane domain (ST) fused to h*R*luc was used to target h*R*luc to the Golgi apparatus. (A) ST–h*R*luc-YFP co-localized with the *cis*-Golgi marker α-mannosidase-CFP. Scale bar=20 µm. (B) ST–h*R*luc and h*R*luc activities in transiently expressing *N. benthamiana* crude leaf protein extracts. The silencing suppressor p19 was co-expressed with ST–h*R*luc and h*R*luc constructs and alone is used as a negative control for background luminescence. Error bars represent 95% confidence interval, *n*=3. Log_10_(RLU); relative luminescence units transformed to Log_10_. (This figure is available in colour at *JXB* online.)

The functionality of h*R*luc within the Golgi lumen, never previously demonstrated *in planta*, was determined. The 52 amino acid residues from the N-terminus of ST, including the 17 residue *trans*-membrane region and 9 cytosolic residues, have previously been shown to be sufficient for Golgi localization (Boevink *et al.*, 1998). Notably the C-terminus localizes to the Golgi lumen (Søgaard *et al.*, 2012). A fusion protein between the N-terminal domain of ST and h*R*luc was generated. Transfection of ST–h*R*luc with a C-terminal YFP fusion alongside the Golgi marker α-mannosidase (Nelson *et al.*, 2007), with C-terminal CFP fusion, into *N. benthamiana* confirmed the targeting of h*R*luc to the Golgi apparatus (Fig. 2A). ST–h*R*luc and h*R*luc were transiently expressed in *N. benthamiana* and activity assayed by the conversion of coelenterazine-h to coelenteramide, which undergoes relaxation from an electronically excited state, emitting a photon of blue light. Leaf discs were excised from the infiltrated areas and macerated in an assay buffer (see materials and methods) and this macerate was directly used for the luciferase assay. Robust bioluminescence significantly above background was observed when assaying both ST–h*R*luc and h*R*luc (Fig. 2B). These results demonstrate the suitability of h*R*luc as a reporter in the Golgi lumen. The signal intensity derived from ST–h*R*luc was an order of magnitude lower than that from h*R*luc, which is expected either owing to the generally low abundance of Golgi localized proteins due to the smaller compartment volume of the Golgi apparatus as compared to the cytosol and/or owing to different extractability of the proteins in these compartments.

### Construction of a Gateway-compatible h*R*luc binary vector system

To facilitate the combination of the advantages of *R*luc-PCA with the requirement to examine a multitude of candidate protein interactions within the Golgi apparatus, we sought to create *R*luc-PCA vectors that use Gateway^®^ cloning technology (Life Technologies). Gateway-compatible destination vectors ph*R*luc[F1] (HA-tag) and ph*R*luc[F2] (FLAG-tag) (Supplementary Fig. S1A) were generated that allowed rapid recombination with libraries of genes contained in entry vectors (Lao *et al.*, 2014) and fusion with epitope tags (HA, hemagglutinin; FLAG, the octapeptide DYKDDDDK) for detection of expressed proteins. As the system is Gateway-compatible, genes of interest can easily be cloned and tested in several Gateway-enabled PPI systems including bioluminescence resonance energy transfer (BRET) (Subramanian *et al.*, 2006), FRET (Miyawaki and Tsien, 2000; Siegel *et al.*, 2000), BiFC (Gehl *et al.*, 2009), and the BiFC-based membrane topology analysis (Søgaard *et al.*, 2012). In addition to the Gateway-compatible systems already available, a commercially available split-ubiquitin assay system (DUALmembrane system, Dualsystems Biotech AG, Schlieren, Switzerland) (see details below) was Gateway-enabled for testing membrane-localized PPIs in yeast (Supplementary Fig. S1B).

### Optimization of the *R*luc-PCA system in transient expression in *N. benthamiana*

Initially, highly variable signals of h*R*luc were seen between different infiltrated areas and leaves. As *N. benthamiana* leaves are known to express proteins to different degrees depending on growth stage of the leaves (Cazzonelli and Velten, 2006), the activity of complemented h*R*luc in tissue macerated from manually infiltrated leaves of different ages on the same plant was determined and compared with tissue pooled from the same three leaves (Supplementary Fig. S2A). Expression between leaves was found to be variable within the same plant and therefore the method was refined to pool tissue to reduce variability and ensure reproducibility. Cazzonelli and Velten (2006) found that optimal protein expression occurs between 44–96h post infiltration. To ensure optimal expression, a 72h period was chosen. To determine the optimal integration time for measurement of complemented h*R*luc activity, relative luminescence units (RLU) were measured at half-second intervals for 10 s before and 300 s after addition of coelenterazine-h (Supplementary Fig. S2B). An integration time of 30 s was applied to maximize the integrated signal intensity while minimizing protein degradation. Vacuum infiltration with *Agrobacterium* was also tested, although this resulted in very poor signals, and therefore manual infiltration, pooling of three leaf discs and measurement after 3 d were used for the subsequent experiments.

**Fig. 3.**
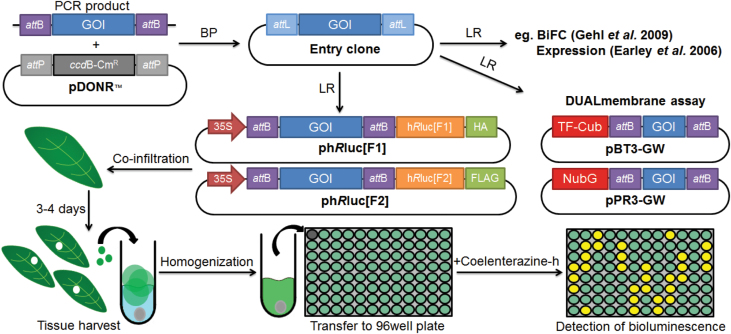
Schematic protocol for *R*luc-PCA. Genes of interest (GOIs) are PCR amplified and recombined into pDONR^TM^/Zeo vector by BP cloning (BP). The GOI-containing entry clone can be recombined by LR cloning (LR) to insert GOI into desired destination vectors, which here is the Gateway-compatible ph*R*luc[F1] and ph*R*luc[F2] belonging to *R*luc-PCA. Alternatively, GOI can be recombined into other destination vectors, e.g. Gateway-compatible DUALmembrane vectors containing the amino terminal ubiquitin fragment (NubG) and the carboxy terminal ubiquitin fragment fused to an artificial transcription factor (TF–Cub). In *R*luc-PCA, ph*R*luc[F1]- and ph*R*luc[F2]-fused GOIs are individually introduced into different *Agrobacterium* and then co-infiltrated into the leaves of *N. benthamiana* in desired combinations to test PPIs. The assay is performed 3–4 d post infiltration by harvesting three leaf discs and transferring to tubes containing 200 µl assay buffer and a chrome ball. Leaf discs are macerated and 100 µl is transferred into a black 96-well plate. Bioluminescence upon addition of coelenterazine-h is monitored in a plate luminometer. (This figure is available in colour at *JXB* online.)

An overview of the developed assay procedure is shown in Fig. 3. Combinations of Agrobacterial strains containing constructs of interest can be made in 24-well plates for convenient handling, with each combination requiring not more than 1ml in volume. In our hands, manual infiltration of 50 combinations, each infiltrated in three different leaves, by one person takes approximately 1–2h, allowing a mid-throughput analysis. This processing time was comparable to that required for a manually performed split-ubiquitin assay in yeast with the same number of samples. After 72h, three leaf discs, each derived from independent infiltrated areas, were excised, pooled, and macerated in 96-well plates with a ball mixer mill. The macerates were then transferred to fresh 96-well plates for luminescence measurement. A microplate reader with high-sensitivity luminescence detector equipped with auto-reagent-injectors was used in this study.

### Establishing a proof of concept for identifying PPIs in the Golgi lumen

To confirm that the *R*luc-PCA was functional in reporting PPIs within the Golgi lumen, cell wall biosynthetic GTs that were previously shown to form complexes in the Golgi apparatus were selected to tested with *R*luc-PCA. GAUT, GAUT7, and ARAD1 were selected as these proteins form hetero- and homodimeric complexes, respectively (Atmodjo *et al.*, 2011; Harholt *et al.*, 2012). These proteins exhibit the canonical type II membrane topology and the C-termini of these proteins are known to be lumenal (Atmodjo *et al.*, 2011; Søgaard *et al.*, 2012). The GAUT1–GAUT7 PPI also allowed the resilience of the system in assaying interactions between membrane-associated proteins and soluble lumenal proteins. Additionally, IRX9, also a Golgi-localizing GT (Brown *et al.*, 2007; Peña *et al.*, 2007), was included as a control to test for non-specific interactions. As IRX9 is known to be involved in hemicellulosic glucuronoxylan synthesis in the secondary cell wall (Brown *et al.*, 2007; Peña *et al.*, 2007), whereas the GAUT1–GAUT7 heterodimer and the ARAD1 homodimer are involved in pectic homogalacturonan (Atmodjo *et al.*, 2011) and arabinan synthesis (Harholt *et al.*, 2006) in the primary cell wall, respectively, we hypothesised that these proteins would not interact. Analysis of the amino acid sequence of IRX9 shows that it has a typical type II membrane topology, indicating that the IRX9 C-terminus is also lumenal (Supplementary Fig. S3).

**Fig. 4.**
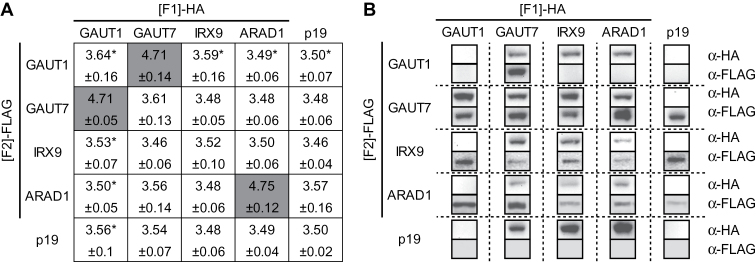
*R*luc-PCA identifies the GAUT1–GAUT7 core-complex and ARAD1–ARAD1 homodimer. (A) Heat map of Log_10_ values of RLU where dark grey denotes statistically significant higher Log_10_ values of RLU above the background level (p19). Statistical analysis was performed on the averages derived from three independent experiments, each consisting of three biological replicates (pools) (see materials and methods). A vector containing the silencing suppressor p19 was co-transfected along with GOI–h*R*luc[F1] and GOI–h*R*luc[F2]. Error represents 95% confidence interval, *n*=3. Asterisk represents extracts where GAUT1 was not detected by immunoblot owing to proteolytic processing and possible degradation (Atmodjo *et al.*, 2011). (B) Immunoblot of expressed proteins probed with anti-HA and anti-FLAG primary antibodies.

Agrobacterial strains harbouring GAUT1, GAUT7, ARAD1, and IRX9 recombined into ph*R*luc[F1] and ph*R*luc[F2] were co-infiltrated into *N. benthamiana* leaves alongside the silencing suppressor p19 (Voinnet *et al.*, 2003). The OD value of each strain was 0.2 whereas that for p19 was 0.05. Binary PPI assays among GAUT1, GAUT7, ARAD1, and IRX9 showed that, after 72h incubation, only tissues expressing the combinations GAUT1-[F1] and GAUT7-[F2] (Log_10_ value: 4.71), GAUT7-[F1] and GAUT1-[F2] (Log_10_ value: 4.71), and ARAD1-[F1] and ARAD1-[F2] (Log_10_ value: 4.75) showed complemented luciferase activity with an order of magnitude higher RLU than those expressing the single halves of h*R*luc or p19 alone (Log_10_ value: 3.50) (Fig. 4A). Immunoblots confirmed that, as expected, soluble GAUT1 was only retained in leaves also expressing its anchor GAUT7. In all other cases, immunoblots confirmed expression of two proteins in extracts testing both positive and negative interactions by *R*luc-PCA, indicating a negative measurement was due to lack of interaction and not lack of expression (Fig. 4B). Measured RLUs of positive complementations were between approximately 16–18 fold higher than that of background demonstrating the robustness of the *R*luc-PCA in discerning positive interactions in the Golgi lumen above non-specific noise. The average RLU from the positive interactions was 2.3%±0.12 of the RLU obtained for the Golgi-localized h*R*luc. Taken together, these results demonstrate that *R*luc-PCA can successfully identify known Golgi PPIs and can distinguish positive PPIs from the background.

Impact of protein overexpression on the bioluminescence complementation was analysed. ARAD1-[F1] and ARAD1-[F2] were co-expressed at equal Agrobacterial OD values ranging from 0.025–0.2. This OD range was chosen because ARAD1 fused to GFP localizes to the Golgi apparatus when infiltrated at the OD value of 0.05, whereas increasing ODs caused mistargeting to the endoplasmic reticulum (Sakuragi *et al.*, 2011). Log_10_ RLU values obtained for all the samples were significantly higher than that of the negative control (p19 only), whereas no significant difference was observed among the samples within the tested OD range (Supplementary Table S2). These results indicate that overexpression of ARAD1 does not increase the bioluminescence signal. Targeting of glycosyltransferases to sub-Golgi compartments can be mediated by protein complex formation, known as “kin recognition”, which functions by forming protein aggregates that are too large to enter transport vesicles (Nilsson *et al.*, 1993). It is plausible that ARAD1 forms homomeric complexes to remain in the Golgi apparatus or in a sub-Golgi compartment and those proteins that were mistargeted to the endoplasmic reticulum owing to overexpression do not form complexes and thus do not contribute to bioluminescence complementation.

In addition, higher OD values (0.2 and 0.1) for ARAD1-[F1] were infiltrated alongside a lower OD value (0.05) for ARAD-[F2] and bioluminescence measured. Log_10_ RLU values of both combinations were significantly higher than that of the negative control but were not significantly different in comparison to the sample where the OD value for the both proteins was 0.2 (*P*-value>0.05) (Supplementary Table S2). This result suggests that the bioluminescence complementation of ARAD1-[F1] and ARAD1-[F2] is independent of the ratio of the expressed protein levels within the range tested.

Finally, a competition assay was performed in which ARAD1-[F1] and ARAD1-[F2] (OD value of 0.1 for each) were co-expressed with a cMyc-tagged ARAD1 as the competitor (OD values of 0, 0.2, and 0.4) (Table 1 and Supplementary Fig. S4). The complemented bioluminescence diminished with increasing concentration of the competitor, demonstrating that the observed bioluminescence complementation is not due to a false positive effect.

**Table 1.** Competition assayARAD1-[F1] and ARAD1-[F2] were co-expressed with ARAD1–cMyc as the competitor at increasing Agrobacterial ODs. p19 was co-expressed in all samples (OD 600nm=0.05). Log_10_ values of RLU were obtained from three biological replicates, errors represents the 95% confidence interval.

Construct		OD	
ARAD1-[F1]	0.1	0.1	0.1
ARAD1-[F2]	0.1	0.1	0.1
35S–ARAD1–cMyc	0	0.2	0.4
Log_10_(RLU)	4.20 ±0.059	4.07 ±0.019	3.96 ±0.011

### *R*luc-PCA among hemicellulosic xyloglucan and xylan biosynthetic enzymes

**Fig. 5.**
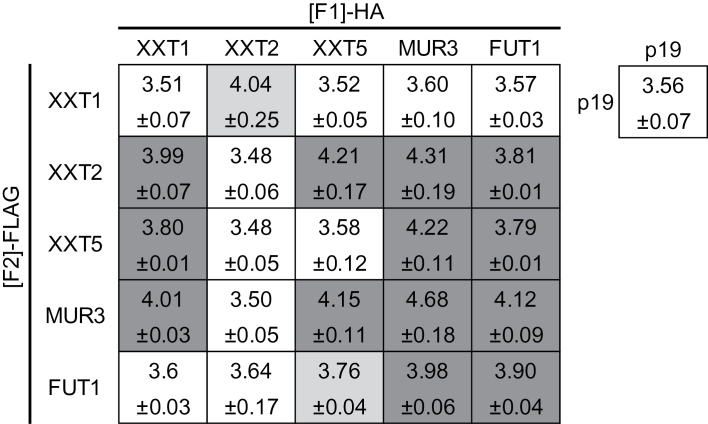
Application of the *R*luc-PCA to test PPIs among XyG biosynthetic enzymes. Three independent experiments, each consisting of three biological replicates (pools) were made (see materials and methods). Generated results are shown as heat map of Log_10_ values of RLU where dark grey denotes samples with all experiments being significantly higher than the background level, whereas light grey denotes samples with two out of three experiments being significantly higher than the background level and white denotes Log_10_ values of RLU of the background p19 infiltrated control in a complementation assay. Statistical analysis was performed on the averages derived from three independent experiments. Plants were co-transfected with Agrobacteria carrying vectors containing silencing suppressor p19, GOI–h*R*luc[F1], and GOI–h*R*luc[F2]. Error represents 95% confidence interval, *n*=3.

*R*luc-PCA coupled with transient expression in *N. benthamiana* was applied to test binary interactions among XyG biosynthetic enzymes: XXT1 (Cavalier and Keegstra, 2006), XXT2 (Cavalier and Keegstra, 2006), XXT5 (Zabotina *et al.*, 2008), MUR3 (Madson *et al.*, 2003; Tamura *et al.*, 2005), FUT1 (Perrin *et al.*, 1999; Perrin *et al.*, 2003), and CSLC4 (Cocuron *et al.*, 2007). Expression of fusion proteins was confirmed by immunoblot analysis (Supplementary Fig. S5), with the exception of CSLC4-[F1] and -[F2], which were not detectable. The background RLU level of *N. benthamiana* expressing p19 was Log_10_ value of 3.56. The lower and upper limits of the range of detected RLU found to be significantly higher than background (p19) were XXT5-[F1] and FUT1-[F2] with a Log_10_ value of 3.76, and MUR3-[F1] and MUR3-[F2] with a Log_10_ value of 4.75, which are approximately 5800 RLU and 56000 RLU, respectively. The tested combination consisting of XXT1 and XXT2, XXT5 and FUT1, XXT5 and MUR3, MUR3 and FUT1, MUR3 and MUR3, FUT1 and FUT1 (Fig. 5) all had statistically significant (*P*<0.05) RLUs above the background level. These positive PPIs were detectable when the [F1] and [F2] tags were swapped in at least five out of six biological replications and therefore were considered as genuine PPIs with high confidence. XXT1 and XXT2 formed PPIs with many other XyG enzymes when they were fused to the [F2] and [F1] tags, respectively, whereas no PPI was formed when the tags were swapped (Fig. 5). XXT1-[F2] and XXT2-[F1] may be improperly folded or the h*R*luc tags are not oriented properly to allow complementation of the luciferase activity. Therefore, the results were interpreted as an indication of PPIs with lower confidence among the following XyG enzymes: XXT1 and XXT5, XXT1 and MUR3, XXT2 and XXT5, XXT2 and MUR3, XXT2 and FUT1. CSLC4 has a topology locating both N- and C-termini to the cytosolic side of the Golgi membrane (Davis *et al.*, 2010), whereas the other tested proteins are Golgi-localized type II membrane proteins that have their C-termini within the Golgi lumen (Søgaard *et al.*, 2012). This caused the split h*R*luc tags to be located on opposite faces of the membrane rendering complementation of h*R*luc impossible when testing CSLC4 against Golgi-localized type II membrane proteins, and such were not tested.

There is evidence from wheat that proteins from GT43, GT47, and GT75 form a higher order complex in arabinoxylan synthesis (Zeng *et al.*, 2010). It has previously been speculated that the enzymes involved in synthesis of the β-1,4-linked xylan backbone, namely IRX9 and IRX14 of GT43, and IRX10 of GT47, may too form PPIs in *Arabidopsis* (Brown *et al.*, 2009; Faik *et al.*, 2014; Oikawa *et al.*, 2013). We carried out *R*luc-PCA amongst these proteins and their homologues IRX9-L, IRX14-L, and IRX10-L. Luminescence above background was not detected for any combination of these enzymes, indicating no direct PPIs occurring amongst the xylan biosynthetic GTs under the conditions tested (Supplementary Fig. S6).

### Split-ubiquitin assay of XyG and xylan biosynthetic enzymes

**Fig. 6.**
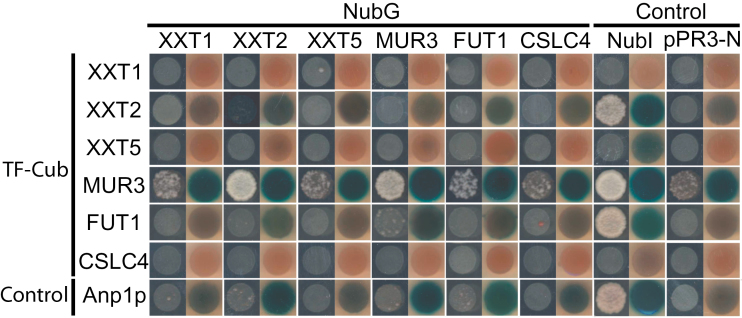
Split-ubiquitin assay used to detect PPIs among XyG biosynthetic proteins. Transformed yeast containing the indicated combinations of TF–Cub and NubG fused proteins were spotted in an OD_546_ of 1.5 and up to 1000× dilution on SD-His-Leu and SD-His-Leu-Trp-Ade plates. Growth on SD-His-Leu-Trp-Ade plates indicates a positive interaction. X-Gal assay performed on growing yeast on SD-His-Leu is a test for β-galactosidase activity, a reporter for interaction upon blue colour formation, Ost1p–NubI (NubI) and pPR3-N test for the functionality and random interaction of the Cub-fused proteins, respectively. The type II membrane protein TF–Cub–Anp1p tests for random interaction among NubG-fused proteins. Consensus of three biological replicates is shown. (This figure is available in colour at *JXB* online.)

For comparison, the split-ubiquitin assay in yeast was used to test PPIs among the XyG and xylan biosynthetic enzymes. The original split-ubiquitin system requires the synthetic TF to be fused to the C-terminus of Cub and to be localized to the cytosol to be cleaved off by ubiquitin-specific proteases and to initiate transcriptional activation of reporter genes in the nucleus (Stagljar *et al.*, 1998). Because many GTs, including those described in this study, have type II membrane topology, C-terminal tagging would result in the Cub-TF to localize to the Golgi lumen; thus no complementation can occur. Recently a modified yeast split-ubiquitin assay system was made commercially available (Dualsystems Biotech AG, Schlieren, Switzerland), allowing the fusion of the TF at the N-terminus of Cub (TF–Cub); thus the cytosol-localized N-termini of type II membrane proteins. Here, the modified split-ubiquitin assay integrating the Gateway cloning system (see above) was used with a series of controls. Ost1p–NubI was used, where Ost1p, a yeast type I membrane protein localized to the endomembrane system, is fused to NubI, a wild-type variant of Nub that forms stable interaction with Cub. This fusion protein is routinely used to test the functionality of the Cub fusion proteins as a positive control (Stagljar *et al.*, 1998). The empty plasmid, pPR3-N, was used as a negative control to assess auto-activation by the Cub fusion proteins. Anp1p is a Golgi-localized enzyme of yeast involved in N-glycan biosynthesis and was fused to TF–Cub in this study to generate Cub–Anp1p (TF–Cub–Anp1p) as a control to assess random interaction by NubG-fused proteins of interest. TF-Cub-fused XXT1, XXT5, and CSLC4 were found to be non-functional because no growth was found when paired with NubI-fused Ost1p (Fig. 6). Consistently, no PPI involving these proteins was detected. TF-Cub-fused XXT2 was functional but did not form reproducibly detectable PPIs (Fig. 6). In contrast, the TF-Cub-fused MUR3 was found to be functional with only a limited degree of auto-activation (Supplementary Fig. S7), and it showed a significantly high degree of growth when paired with NubG-fused XXT2 and MUR3. TF-Cub-fused FUT1 was also functional but it only showed a limited growth and β-galactosidase activity when paired with NubG-fused MUR3, suggesting an interaction with low confidence. These results indicate that under the conditions tested the split-ubiquitin assay detected PPIs between MUR3 and XXT2, MUR3 and MUR3, and with MUR3 and FUT1.

Of the xylan biosynthetic proteins, when expressed alongside NubI-fused Ost1p, only IRX14 and IRX14-L were demonstrated to be functional TF-Cub fusions (data not shown). The lack of functionality of the majority of the xylan backbone-related GTs under test meant that this line of investigation was not furthered.

### Comparison of XyG and xylan PPIs detected by *R*luc-PCA, the split-ubiquitin assay, and BiFC.

Results obtained in this study and in the previous study by Chou *et al.* (2012), which applied BiFC in *Arabidopsis* protoplasts combined with co-immunoprecipitation of recombinant proteins expressed in *E. coli*, were used for a comparison of the three binary PPI assays for the plant Golgi-localizing proteins involved in XyG biosynthesis (Table 2). Of 10 combinations tested by BiFC in *Arabidopsis* protoplasts and by co-immunoprecipitation of recombinant proteins expressed in *E. coli*, 7 PPIs were observed (Chou *et al.*, 2012). In the study presented here, of the 21 tested, *R*luc-PCA detected 11 PPIs. *R*luc-PCA successfully confirmed three of the PPIs previously detected by Chou *et al.* (2012), XXT1 and XXT2, XXT1 and XXT5, and XXT2 and XXT5, whereas it did not detect homooligomerization of XXT2 and XXT5. The lack of homomeric complementation by XXT2 is likely to be due to the aforementioned improper function of XXT2-[F1], whereas the lack of homomeric complementation by XXT5 is not readily reconciled. It is possible that XXT5 forms a transient interaction that occurs in a kiss-and-go manner, where the proteins are mainly in monomeric form and the complexes form only in a small fraction of time and/or with forces that are too weak to maintain the complex during the sample preparation. Similarly to co-immunoprecipitation, *R*luc-PCA would not be able to generate sufficiently high signals for such interactions. Alternatively, XXT5 may form a transient homomeric association when overexpressed, which could be detectable by BiFC owing to irreversibility of the reporter reconstitution. Aside from the previously reported interactions, *R*luc-PCA identified seven novel PPIs among XyG biosynthetic enzymes: XXT1 and MUR3, XXT2 and MUR3, XXT2 and FUT1, XXT5 and MUR3, XXT5 and FUT1, MUR3 and MUR3, and FUT1 and FUT1. Heterooligomerization of XXT2 and MUR3, and XXT2 and FUT1 have previously been implicated by Zabotina (2012). During the preparation of this manuscript, Zabotina and colleagues have identified heterooligomerization of XXT2 and FUT1, XXT5 and FUT1, MUR3 and FUT1 and homooligomerization of FUT1 by using BiFC and co-immunoprecipitation (personal communication), corroborating our results. Furthermore, PPIs between XXT2 and MUR3, MUR3 and FUT1, and MUR3 itself were verified by split-ubiquitin assay in yeast as described below.

**Table 2.** Comparison of the results obtained by Rluc-PCA, the split-ubiquitin assay (Split-Ub), and bimolecular fluorescence complementation (BiFC)/co-immunoprecipitation (Co-IP) (Chou *et al.*, 2012)0, 1, and 2, indicate no PPI, a PPI with low confidence, and a PPI with high confidence, respectively. nt indicates not tested or not testable owing to non-functional or non-expressed proteins. POI, protein of interest.

Combination		*R*luc-PCA	Split-Ub	BiFC/Co-IP
POI 1	POI 2			
XXT1	XXT1	0	Nt	0
	XXT2	2	Nt	2
	XXT5	1	Nt	1
	MUR3	1	0	nt
	FUT1	0	0	nt
	CSLC4	nt	Nt	0
XXT2	XXT2	0	Nt	2
	XXT5	1	0	2
	MUR3	1	2	nt
	FUT1	1	0	nt
	CSLC4	nt	0	0
XXT5	XXT5	0	Nt	1
	MUR3	2	0	nt
	FUT1	2	0	nt
	CSLC4	nt	Nt	2
MUR3	MUR3	2	2	nt
	FUT1	2	1	nt
	CSLC4	nt	0	nt
FUT1	FUT1	2	0	nt
	CSLC4	nt	0	nt
CSLC4	CSLC4	nt	Nt	2

Recently, binary interactome analysis among 3286 membrane and signalling proteins from *Arabidopsis* were carried out (Jones *et al.*, 2014) using the mating-based split-ubiquitin system (Obrdlik *et al.*, 2004), wherein the reporters (Cub–TF and NubG) were fused at the C-termini of the tested proteins. As mentioned above, C-terminal tagging of type II membrane proteins renders the Cub and NubG fragments to be situated inside the Golgi lumen, thereby making them non-functional and this is reflected in the analysis; XXT5 and FUT1, fused to Cub–TF were initially represented in the interactome analysis but were excluded from the analysis owing to “bad topology”, whereas NubG-fusions of XXT5 and FUT1 were still included in the screen, but no PPI involving these proteins was identified. The yeast two-hybrid system was also used to construct an *Arabidopsis* interactome map (*Arabidopsis* Interactome Mapping Consortium, 2011). The yeast two-hybrid system relies on reconstitution of a functional TF followed by transcriptional activation of reporter gene expression in the nucleus. Poor representation of membrane integrated GTs in the interactome by the yeast two-hybrid system is expected, because the system requires the relocation of the assemblage of the reconstituted TF fused to POIs into the nucleus for reporter genes to be transcriptionally activated. GAUT1, GAUT7, XXT2, FUT1, and IRX10-L were represented in the total of approximately 8000 open reading frames screened. The only PPI detected involving these proteins was between FUT1 and At4G19030, a putative aquaporin (AT-NLM1) that formed PPI with 127 other proteins, suggesting false-positive PPIs. The split-ubiquitin assay conducted in this study, using N-terminal tagging, successfully identified heterooligomerization of XXT2 and MUR3, and MUR3 and FUT1, and homooligomerization of MUR3, lending further support to the newly identified PPIs in XyG biosynthesis by *R*luc- PCA. Yet, it is evident that the same assay grossly underestimated the XyG PPIs, and only detected three PPIs in total. This is in large part due to non-functionality of the expressed proteins.

Xylan biosynthetic enzymes of GT43 and GT47 did not form detectable PPIs (Supplementary Fig. S6). This was unexpected as it has been demonstrated that a higher order complex involving members of GT47 and GT43 is formed in wheat (arabino)xylan synthesis which promotes synthesis of the β(1,4) xylan backbone (Zeng *et al.*, 2010) and because indirect evidence support the existence of xylan synthase complexes in *Arabidopsis* (Brown *et al.*, 2009; Faik *et al.*, 2014). It remains possible that these proteins do not form complexes and act independently on the oligosaccharide substrates. It is also possible that additional proteins are required to form complexes including these enzymes. Nevertheless, the lack of interactions in xylan biosynthesis underpins the specificity of PPIs detected by *R*luc-PCA.

Based on Table 2, we hypothesize that there exists a XyG biosynthetic PPI network. In *Arabidopsis* XyG biosynthesis, CSLC4 is thought to catalyse the synthesis of β-1,4-linked glucan backbone (Cocuron *et al.*, 2007), which is decorated with the side-chain α-1,6-xylosyl residues by XXT1, XXT2, and XXT5 (Cavalier and Keegstra, 2006; Cavalier *et al.*, 2008; Zabotina *et al.*, 2008) and can be further substituted with β-1,2-galactosyl residue by MUR3 and XYLOGLUCAN L-SIDE CHAIN GALACTOSYLTRANSFERASE POSITION2 (XLT2) and α-1,2-fucosyl residues by FUT1 (Faik *et al.*, 2000; Jensen *et al.*, 2012; Madson *et al.*, 2003; Scheller and Ulvskov, 2010; Zabotina *et al.*, 2008). In this study, PPIs among XXT1, XXT2, XXT5, MUR3, and FUT1 were tested and nearly all these side-chain forming enzymes were found capable of forming PPIs with each other, which raises a possibility that XXT1, XXT2, XXT5, MUR3, and FUT1 form a side-chain forming complex(es). It is noteworthy that XXT5 is thus far the only side-chain forming enzyme that has been shown to form PPI with CSLC4 (Chou *et al.*, 2012). Also noteworthy is that MUR3 was previously identified as KATAMARI1, which is required for the proper functioning of actin in maintaining the endomembrane organization in *Arabidopsis* through protein complex formation with actin (Tamura *et al.*, 2005). Based on these previous observations and the results obtained in the present study, we speculate (i) that XXT5 tethers the side-chain forming complex(es) to the backbone synthesis by CSLC4 to form a XyG biosynthetic complex(es) and (ii) that this complex(es) is further anchored to actin filaments via MUR3 to ensure proper actin organization that is required for the secretion of polysaccharides from the Golgi stacks to the cell wall (Blancaflor, 2002; Hu *et al.*, 2003; Tamura *et al.*, 2005). The hypothesis of XXT5 tethering is consistent with the phenotypes of various xyloglucan mutants as previously reported (Zabotina *et al.*, 2012). The authors concluded that XXT5 cannot add the xylose residues on its own and raised a possibility that the function of XXT5 is to maintain the integrity of a synthetic complex involved in xyloglucan biosynthesis rather than to function as a xylosyltransferase. Although this possibility is yet to be substantiated, our results lend support to it. Based on the physiological data by Zabotina *et al.* (2012) and our results, we speculate that the exact protein composition of xyloglucan complexes is probably variable depending on tissues types; for instance in seedling roots XXT5 is largely dispensable, whereas in hypocotyl XXT5 plays a major role in determining and/or maintaining the composition of xyloglucan biosynthetic complex(es).

## Conclusions

The results presented in the current study demonstrate that *R*luc-PCA adapted for use in transient expression in *N. benthamiana* allows easy, rapid, and mid-throughput screening of PPIs among Golgi-resident membrane proteins. Integration of Gateway technology enables versatile choice of additional PPI systems (BiFC and split-ubiquitin assay in yeast) to be applied. The system can be readily used for studies of PPIs in the lumen of the endomembrane system as well as in the cytosol.

## Supplementary data

Supplementary data are available at *JXB* online

Figure S1. Maps of destination vectors produced in this study

Figure S2. Refinement of *R*luc-PCA parameters

Figure S3. Prediction of IRX9 protein topology

Figure S4. Immunoblot of competition assay

Figure S5. Immunoblot of XyG proteins

Figure S6. Application of *R*luc-PCA to test xylan related PPIs

Figure S7. Random interaction of MUR3-bait in split-ubiquitin assay

Table S1. Primers sequences used in this study

Table S2. OD dependency assay

Supplementary Data

## Abbreviations:

BiFC: bimolecular fluorescence complementation
Cub: carboxy terminal fragment of ubiquitin
FRET: Förster resonance energy transfer
GOI: gene of interest
GT: glycosyltransferase
h*R*luc: human-codon optimized *Renilla reniformis* luciferase
Nub: amino terminal fragment of ubiquitin
POI: protein of interest
PPI: protein–protein interaction
RLU: relative luminescence unit
*R*luc: *Renilla reniformis* luciferase
*R*luc-PCA: *Renilla* luciferase complementation assay
ST: Rat sialyltransferase
TF: transcription factor
XyG: xyloglucan.
