# Supplementary material for: A reversible Renilla luciferase protein complementation assay for rapid identification of protein–protein interactions reveals the existence of an interaction network involved in xyloglucan biosynthesis in the plant Golgi apparatus
Source: J Exp Bot. 2014 Oct 18;66(1):85–97. doi: 10.1093/jxb/eru401 (PMC4265154; doi:10.1093/jxb/eru401)
Supplement: Supplementary Data [file supp_66_1_85__index.html]

A reversible Renilla luciferase protein complementation assay for rapid identification of protein–protein interactions reveals the existence of an interaction network involved in xyloglucan biosynthesis in the plant Golgi apparatus — A reversible Renilla luciferase protein complementation assay for rapid identification of protein–protein interactions reveals the existence of an interaction network involved in xyloglucan biosynthesis in the plant Golgi apparatus — Supplementary Data 

# A reversible *Renilla* luciferase protein complementation assay for rapid identification of protein–protein interactions reveals the existence of an interaction network involved in xyloglucan biosynthesis in the plant Golgi apparatus

## Supplementary Data

Data files

**Files in this Data Supplement:**

- Supplementary Data - Supplementary Data
