# Supplementary material for: A reversible Renilla luciferase protein complementation assay for rapid identification of protein–protein interactions reveals the existence of an interaction network involved in xyloglucan biosynthesis in the plant Golgi apparatus
Source: J Exp Bot. 2014 Oct 18;66(1):85–97. doi: 10.1093/jxb/eru401 (PMC4265154; doi:10.1093/jxb/eru401)
Supplement: Supplementary Data [file supp_eru401_jexbot129619_file001.pdf]

Supplementary material

Title:

A reversible *Renilla* luciferase protein complementation assay for rapid identification of protein-protein interactions revealed the existence of an interaction network involved in xyloglucan biosynthesis in the Golgi apparatus in plant.

Authors:

Christian Have Lund, Jennifer R. Bromley, Anne Stenbæk, Randi E. Rasmussen, Henrik V. Scheller & Yumiko Sakuragi.

Figure S1. Maps of destination vectors produced in this study
Figure S2. Refinement of *R*luc-PCA parameters
Figure S3. Prediction of IRX9 protein topology
Figure S4. Immunoblot of competition assay
Figure S5. Immunoblot of XyG proteins
Figure S6. Application of *R*luc-PCA to test xylan biosynthetic enzymes related PPIs
Figure S7. Random interaction of MUR3-bait in split-ubiquitin assay
Table S1. Primers sequences used in this study
Table S2. OD dependency assay

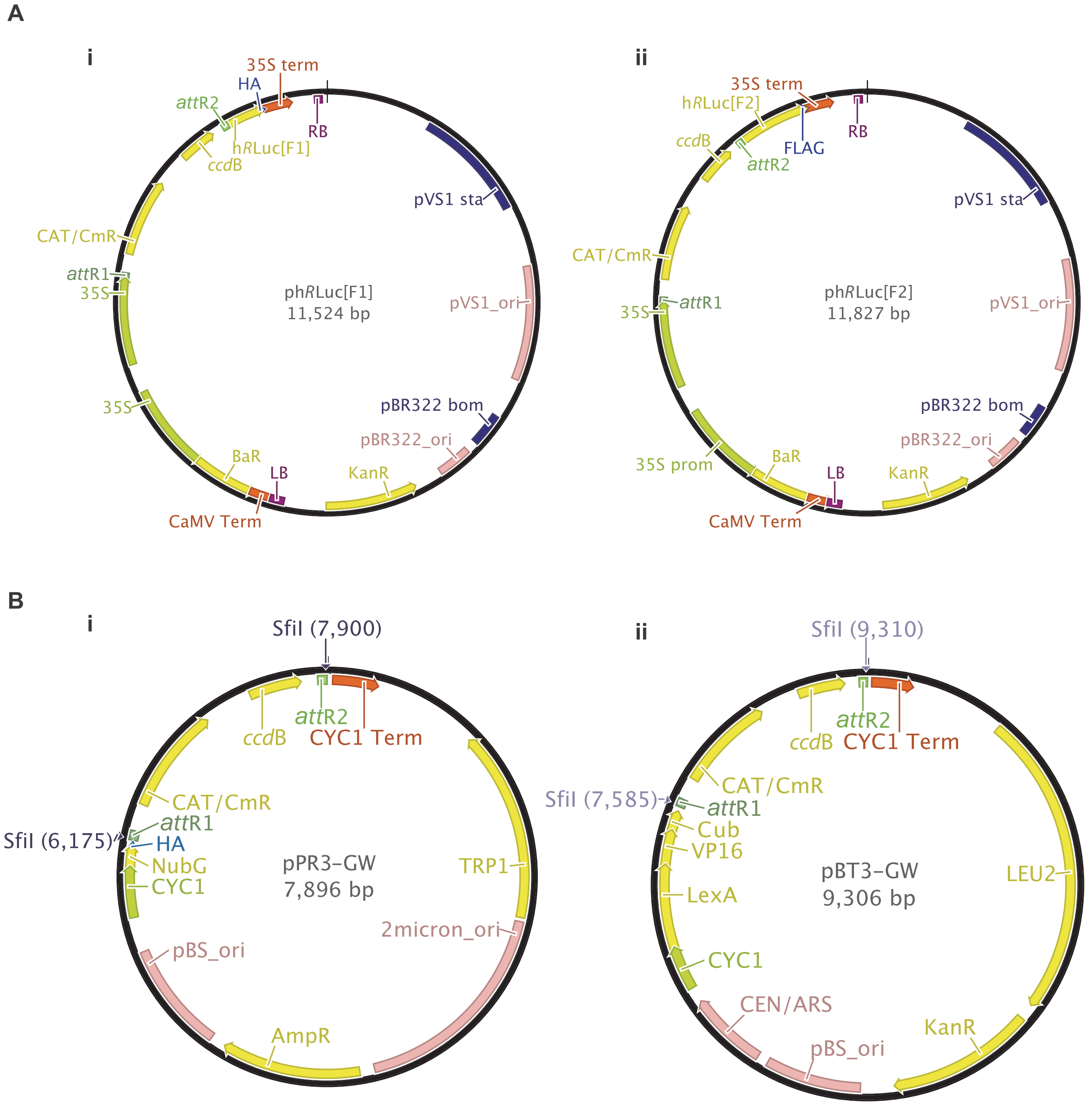

Fig. S1. Maps of destination vectors produced in this study **A.** *R*luc-PCA vectors i. ph*R*luc[F1] and ii. ph*R*luc[F2] **B.** Split-ubiquitin assay vectors i. pBT3-GW ii. pPR3-GW.

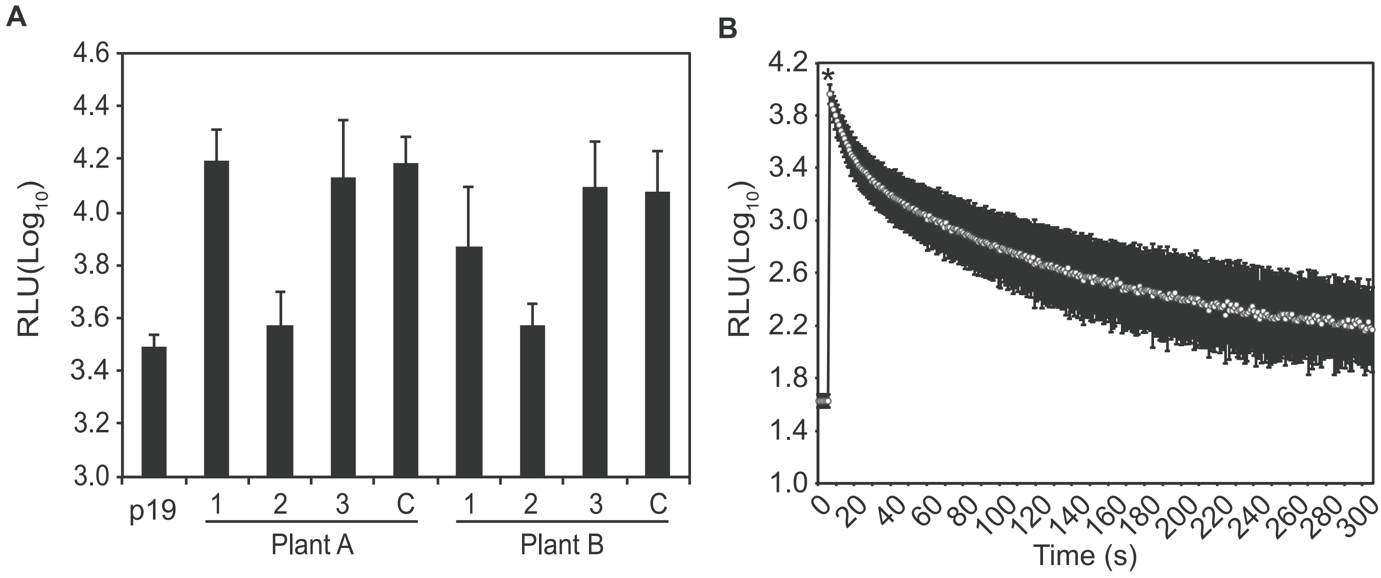

Fig. S2. Refinement of *R*luc-PCA parameters. Complemented h*R*luc activities measured for *N. benthaminana* transiently transfected with a positive control PPI pair (hRluc-GAUT1-[F1]/hRluc-GAUT7-[F2]). **A.** Assayed from extracts of individual and pooled leaves from the same plant. Numbers represent samples taken from individual leaves, C represents a pooled sample with disks from each leaf. Bioluminescence, RLU(Log_{10}), was measured for 30 s for each sample. Error bars represent 95% confidence interval, n=3. **B.** Time course of detected bioluminescence, RLU(Log_{10}), after addition of coelenterazine-h to 10 µM (addition marked with *). Each data point represents bioluminescene detected during a period of 0.5 s. Error bars represent 95% confidence interval, n=3.

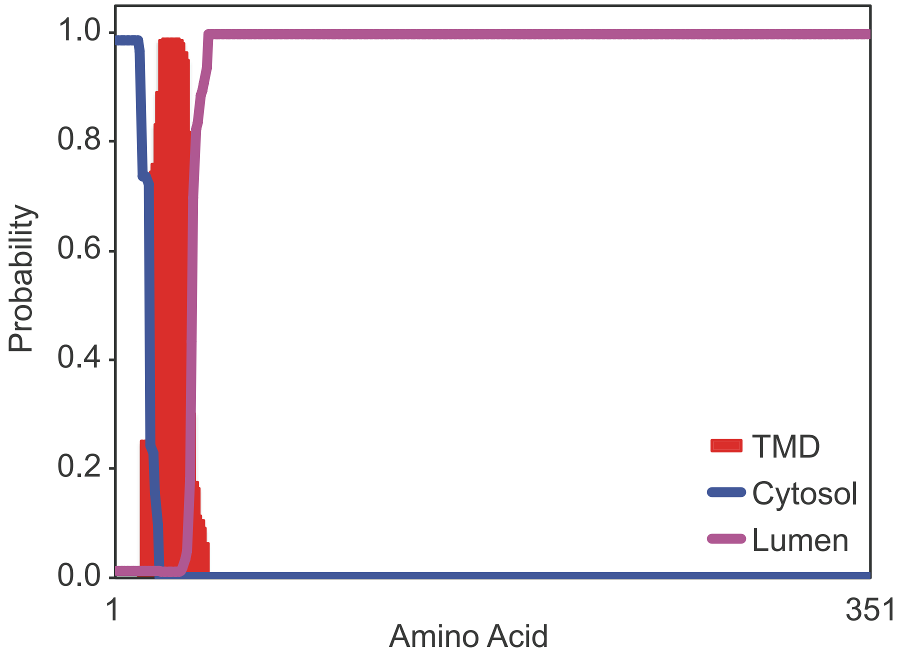

Fig. S3. Prediction of IRX9 protein topology. Topology prediction of IRX9 (At2g37090.1) from TmHMM2.0 (http://www.cbs.dtu.dk/services/TMHMM-2.0/) (Krogh *et al*., 2001). This is consistent with those predicted by servers consolidated on ARAMEMNON (http://aramemnon.botanik.uni-koeln.de/index.ep) (Schwacke *et al*., 2003).

Construct	OD		
35S-ARAD1-cMyc	0	0.2	0.4
a-cMyc			

Fig. S4. Immunoblot of competition assay. ARAD1-cMyc was co-expressed as the competitor at increasing Agrobacterial ODs with ARAD1-F1 and ARAD1-F2. p19 was co-expressed in all samples (OD 600nm = 0.05). Immunoblot detection of ARAD1-cMyc was performed with monoclonal anti-cMyc antibody. Approximate size of ARAD1-cMyc is approximately 53 kDa. Bioluminescence signals for the corresponding samples are presented in Table 1.

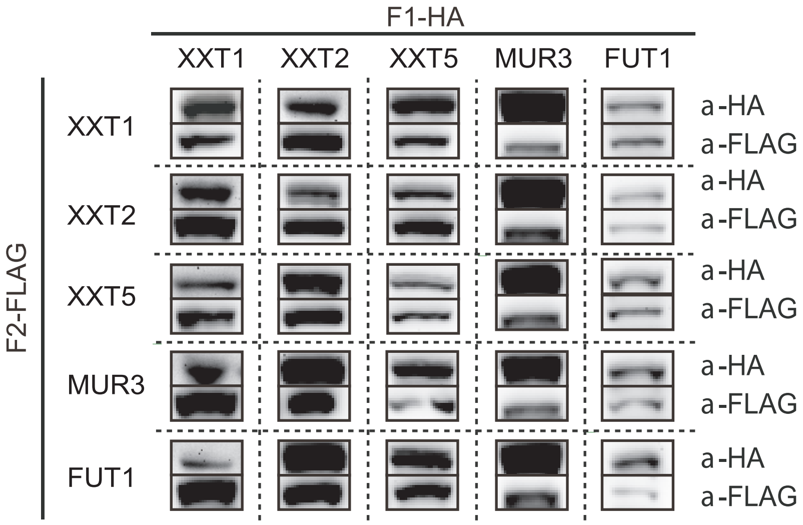

Fig. S5. Immunoblot of XyG proteins. Detection of protein expression in macerated tobacco leaf discs using polyclonal HA and monoclonal FLAG antibodies. Approximate sizes of [F1]-HA tagged proteins: ph*R*luc-XXT1-[F1], 68 kDa; ph*R*luc-XXT2-[F1], 68 kDa; ph*R*luc-XXT5-[F1], 67 kDa; ph*R*luc-MUR3-[F1], 86 kDa; ph*R*luc-FUT1-[F1], 78 kDa; and [F2]-FLAG tagged proteins ph*R*luc-XXT1-[F2], 79 kDa; ph*R*luc-XXT2-[F2]: 79 kDa; ph*R*luc-XXT5-[F2], 77 kDa; ph*R*luc-MUR3-[F2], 97 kDa; ph*R*luc-FUT1-[F2], 89 kDa.

		F1-HA							
		IRX9	IRX9-L	IRX10	IRX10-L	IRX14	IRX14-L	ARAD1	p19
F2-FLAG	IRX9	3.50 ±0.29	3.61 ±0.23	3.79 ±0.43	3.60 ±0.12	3.68 ±0.18	3.49 ±0.4	3.47 ±0.33	3.50 ±0.44
	IRX9-L	3.52 ±0.25	3.59 ±0.14	3.70 ±0.29	3.69 ±0.29	3.69 ±0.14	3.67 ±0.23	3.75 ±0.18	3.45 ±0.33
	IRX10	3.48 ±0.09	3.71 ±0.08	3.68 ±0.16	3.05 ±0.99	3.74 ±0.20	3.73 ±0.25	3.63 ±0.08	3.53 ±0.20
	IRX10-L	3.66 0.08	3.63 ±0.07	3.69 ±0.17	3.69 ±0.12	3.63 ±0.22	3.47 ±0.10	3.64 ±0.28	3.61 ±0.20
	IRX14	3.73 ±0.24	3.65 ±0.14	3.67 ±0.02	3.71 ±0.26	3.65 ±0.28	3.70 ±0.12	3.57 ±0.17	3.71 ±0.06
	IRX14-L	3.89 ±0.27	3.52 ±0.11	3.67 ±0.23	3.66 ±0.09	3.61 ±0.12	3.44 ±0.15	3.67 ±0.19	3.33 ±0.28
	ARAD1	3.59 ±0.05	3.56 ±0.18	3.62 ±0.10	3.67 ±0.15	3.75 ±0.12	3.62 ±0.08	4.72 ±0.10	3.57 ±0.16
	p19	3.62 ±0.18	3.59 ±0.30	3.58 ±0.29	3.30 ±0.57	3.62 ±0.29	3.64 ±0.16	3.34 ±0.30	3.58 ±0.28

Fig. S6. Application of *R*luc-PCA to test xylan biosynthetic enzymes related PPIs. *R*luc-PCA showed no PPIs amongst GT43 and GT47 proteins involved in xylan backbone synthesis. ARAD1 was included as a positive control. Heat map of Log_{10} values of RLU where dark grey denotes statistically significant higher Log_{10} values of RLU above the background level and white the Log_{10} values of RLU of the background p19 infiltrated control. A vector containing the silencing suppressor p19 was co-transfected along with GOI-h*R*luc[F1] and GOI-h*R*luc[F2]. Error represents 95% confidence interval, n=3.

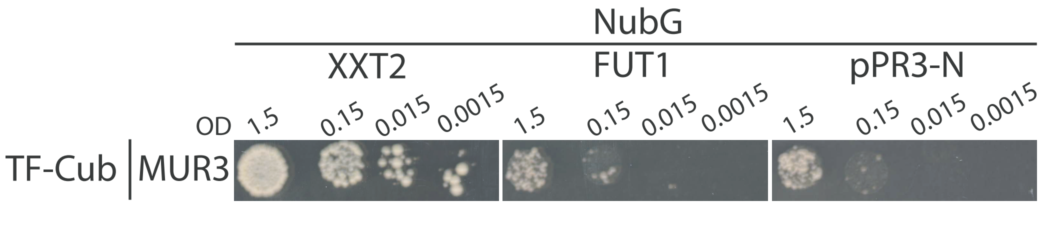

Fig. S7. Random interaction of TF-Cub-MUR3 in split-ubiquitin assay. True vs. random interaction illustrated by the true interaction of TF-Cub-MUR3 and NubG-XXT2 due to extensive growth throughout the dilution series and the random interactions between TF-Cub-MUR3 and NubG-FUT1 due to similar growth of random interaction test (TF-Cub-MUR3 against empty pPR3-N vector) with almost exclusively growth in undiluted spot.

Table S1. Primers sequences used in this study

Primer	Sequence (5'-3')
Cloning and relocalising h*R*luc	
USERF1 F	GGCTTAAUATGGCTTCCAAGGTGTACGA
USERF1 R	ATTTTCUTTGGAAGGTTCAGCAGCTC
USERF2 F	AGAAAAUCATCTTTGTGGGCCACGAC
USERF2 R	GGTTTAAUTTACTGCTCGTTCTTCAGCA
*att*B1Luc F	GGGGACAAGTTTGTACAAAAAAGCAGGCTTCACCATGGCTTCCA AGGTGTACGA
*att*B2Luc R	GGGGACCACTTTGTACAAGAAAGCTGGGTCCTGCTCGTTCTTCA GCACG
*att*B1ST F	GGGGACAAGTTTGTACAAAAAAGCAGGCTTCACCATGATTCATAC CAACTTGAAGAAAAAG
LucST R	AAGCCATGGCCACTTTCTC
STLuc F	AGTGGCCATGGCTTCCAA
h*R*luc PCA vector construction	
USERGW F	GGCTTAAUACAAGTTTGTACAAAAAAGCTGAA
LucF1GW R	GTACACCTTGGAAGCACCTACCACTTTGTACAAGAAAGCTGAAC
GWLucF1 F	CTTGTACAAAGTGGTAGGTGCTTCCAAGGTGTACGACCCCGAG
USERLucF1HA R	GGTTTAAUTCAAGCGTAATCTGGAACAT
LucF2GW R	GATGATTTTCTTTGGACCTACCACTTTGTACAAGAAAGCTGAAC
GWLucF2 F	CTTGTACAAAGTGGTAGGTCCAAAGAAAATCATCTTTGTGGGC
USERLucF2FL R	GGTTTAAUTCATTTGTCGTCATCGTCTTTGTAGTCGGACCCACCA CCTCCAGAGC
Gateway enabling DUALmembrane vectors	
SfiGW F	GGCCATTACGGCCACAAGTTTGTACAAAAAAGCTGAAC
SfiGWSTOP R	GGCCAAGGAGGCCTCATACCACTTTGTACAAGAAAGCT
Production of entry vectors by BP recombination	
GAUT1 F	GGGGACAAGTTTGTACAAAAAAGACGGCTATGGCGCTAAAGCGA GGGCT
GAUT1 R	GGGACCACTTTGTACAAGAAAGCTGGGTGTTCATGAAGGTTGCA ACGAC
GAUT7 F	GGGGACAAGTTTGTACAAAAAAGACGGCTATGAAAGGCGGAGGC GGTGG
GAUT7 R	GGGACCACTTTGTACAAGAAAGCTGGGTGAGGATTCACGTTACA GTCAC
ARAD1 F	GGGGACAAGTTTGTACAAAAAAGCAGGCTTCACCATGGCGCGTA AATCTTCC
ARAD1 R	GGGGACCACTTTGTACAAGAAAGCTGGGTCAATGGAAGTGATAA GACCGGTTT

IRX9 F	GGGGACAAGTTTGTACAAAAAAGACGGTCACCATGGGATCTCTAGAGAGATCAAAG
IRX9 R	GGGACCACTTTGTACAAGAAAGCTGGGTCGGTGCTTAAACGTGTTCTTGTGGGAAA
XXT5 F	GGGGACAAGTTTGTACAAAAAAGACGGTCACCATGGGTCAAGATGGTTCGCC
XXT5 R	GGGACCACTTTGTACAAGAAAGCTGGGTCGTTCTGTGGTTTGGTTTCC
MUR3 F	GGGGACAAGTTTGTACAAAAAAGACGGTCACCATGTTTCCAAGGGTTTCTATGAGG
MUR3 R	GGGACCACTTTGTACAAGAAAGCTGGGTCCTGTGTCTTATCTCTCTGC
FUT1 F	GGGGACAAGTTTGTACAAAAAAGACGGTCACCATGGATCAGAATTCGTACAG
FUT1 R	GGGACCACTTTGTACAAGAAAGCTGGGTCTACTAGCTTAAGTCCCCAGC
Cloning into DUALmembrane vectors via SfiI restriction site	
XXT1 F	ATTAACAAGGCCATTACGGCCATGATAGAGAAGTGTATAGG
XXT1 R	AACTGATTGGCCGAGGCGGCCTCACGTCGTCGTCGTACTAAGC
XXT2 F	ATTAACAAGGCCATTACGGCCATGATTGAGAGGTGTTTAGG
XXT2 R	AACTGATTGGCCGAGGCGGCCTCAAACTTGATTGGTTTGTACC
XXT5 F	ATTAACAAGGCCATTACGGCCATGGGTCAAGATGGTTCGCC
XXT5 R	AACTGATTGGCCGAGGCGGCCCTAGTTCTGTGGTTTGGTTTCC
CSLC4 F	ATTAACAAGGCCATTACGGCCATGGCTCCAAATTCAGTAGC
CSLC4 R	AACTGATTGGCCGAGGCGGCCCTAGCTGATCTGTTCTCCGATC
MUR3 F	ATTAACAAGGCCATTACGGCCATGTTTCCAAGGGTTTCTATGAGG
MUR3 R	AACTGATTGGCCGAGGCGGCCTCACTGTGTCTTATCTCTCTGC

Table S2. OD dependency assay. F1 and F2-tagged ARAD1 were co-infiltrated in different OD's along with infiltration of p19 in all samples to investigate the dependency of OD in relation to *R*luc-PCA.

	OD of ARAD1-F1	OD of ARAD1-F2	Log_{10}(RLU)	P value vs. p19	P value Vs. Pair 1
Pair 1	0.2	0.2	3.86 ±0.02	<0.001	
Pair 2	0.1	0.1	3.92 0.03	<0.001	>0.1
Pair 3	0.05	0.05	3.97 ±0.05	<0.01	>0.1
Pair 4	0.025	0.025	3.78 ±0.03	<0.01	>0.05
Pair 5	0.2	0.05	3.92 ±0.06	<0.01	>0.1
Pair 6	0.1	0.05	3.91 ±0.05	<0.01	>0.1
p19 only	-	-	3.61 ±0.2		
